# Numerical Modeling of the Dynamics of Malaria Transmission in a Highly Endemic Region of India

**DOI:** 10.1038/s41598-019-47212-6

**Published:** 2019-08-15

**Authors:** Ruchi Singh Parihar, Prasanta Kumar Bal, Vaibhav Kumar, Saroj Kanta Mishra, Sandeep Sahany, Popat Salunke, Sushil Kumar Dash, Ramesh Chand Dhiman

**Affiliations:** 1 Centre for Atmospheric Sciences, Indian Institute of Technology Delhi, New Delhi, India; 2 National Institute of Malaria Research (ICMR), New Delhi, India

**Keywords:** Environmental health, Malaria

## Abstract

Using a dynamical model (VECTRI) for malaria transmission that accounts for the influence of population and climatic conditions, malaria transmission dynamics is investigated for a highly endemic region (state of Odisha) in India. The model is first calibrated over the region, and subsequently numerical simulations are carried out for the period 2000–2013. Using both model and observations we find that temperature, adult mosquito population, and infective biting rates have increased over this period, and the malaria vector abundance is higher during the summer monsoon season. Regionally, the intensity of malaria transmission is found to be higher in the north, central and southern districts of Odisha where the mosquito populations and the number of infective bites are more and mainly in the forest or mountainous ecotypes. We also find that the peak of the malaria transmission occurs when the monthly mean temperature is in the range of ~28–29 °C, and monthly rainfall accumulation in the range of ~200–360 mm.

## Introduction

The World Health Organization (WHO) estimates that about 36% of the world population is exposed to the risk of malaria. The report also estimates that around 1.3 billion people are at risk of malaria in the South-east Asia region, with about 231 million at high risk^1^. Malaria has a wide distribution of endemicity that extends from South Asia and South-east Asian countries^2^. According to the WHO report^3^, India accounts for three-quarters of all malaria cases in South-East Asia, with around 85.7% of the population exposed to it. Some of the high malaria burden states in India include Odisha, Jharkhand, Chhattisgarh, Madhya Pradesh, Maharashtra, Gujarat and West Bengal^4^. The central and eastern regions of India report the most number of malaria cases, with the largest number of deaths reported in Odisha^5,6^. Studies undertaken in India on malaria in the context of climate change impact show that in Odisha there may be a reduction in malaria transmission but it may still remain malaria endemic in the near future^7^.

Malaria is an acute parasitic illness caused by *Plasmodium falciparum* (more prevalent in forest and hilly areas) or *Plasmodium vivax* (more prevalent in plains) in India. Many studies related to mosquito population dynamics using various dynamical models have been conducted so far over different parts of the world to improve our understanding of malaria transmission and malaria dynamics. Some of the models are based on the effect of mean rainfall and temperature on the biology of malaria transmission. At the global level, the transmission potential of the malaria mosquito population are being assessed using various malaria models^8–11^ such as MIASMA, MARA, LMM, VECTRI, UMEA etc., and the results project that population are at risk due to malaria transmission in many parts of the world, such as East Africa, central Asia, China, parts of Central and South America, India, northern Australia, Thailand and Malaysia. Specifically, these models project high malaria transmission over the tropics^8^. In the past, burdens of malaria mortality for the West African region have been projected using the MARA model^12^. Complex malaria models have been introduced to simulate the African malaria vectors, designed to explicitly simulate mosquito population dynamics at a local scale^13,14^. LMM, MARA, and MIASMA models consider the effect of rainfall and temperature on the biology of malaria transmission and allow the investigation of climatic suitability for malaria transmission, whereas, the VECTRI model considers the impact of climate, surface hydrology, and population densities on malaria transmission. Inputs concerning surface hydrology are of primary importance in a model of malaria transmission, as they contain information related to the availability of potential breeding sites for vectors and capture the impact of rainfall on the disease dynamics. It is worth noting that the VECTRI model incorporates surface hydrology and is likely to perform better in predicting malaria incidence relative to other models^15^. In this aspect, very limited comprehensive studies are available in India that use this model to predict the dynamics of malaria transmissions and distributions. Although the potential of using VECTRI model to predict the dynamics of malaria transmission has been explored previously in different regions, this model has never been evaluated over India on a local scale.

As per the report from National Vector Borne Disease Control Programme^16^, malaria constituted a source of significant health threat in Odisha, and it was reported that around 26.9% of the total malaria cases in India occurred in Odisha. Moreover, *Anopheles culicifacies* has been reported to be the most potent malaria vector in Odisha that contributes more than 90% of the total malaria cases in the state^17–19^. However, quantifying the role of climate variability in the context of malaria transmission dynamics remains an important problem for Odisha. In this aspect, limited studies have been conducted so far using models to predict the malaria transmission dynamics with respect to various climatic factors (temperature, rainfall) and non-climatic factors (malaria parasites, malaria vectors, human host factors, population density), which favor the malaria transmission. More importantly, in Odisha, studies linking to climate variability and malaria transmission across the various districts are limited.

Hence, the objective of the current study is to investigate the spatio-temporal variability in malaria transmission over Odisha using the VECTRI model driven by various climatic and non-climatic factors. The current parameterization scheme of the model includes the activity of *Anopheles gambiae* vector and the dynamics of *Plasmodium falciparum* parasite^15^, seen in African region. However, *Anopheles gambiae* is absent overthe study region, and the species of concern is *Anopheles culicifacies*. Therefore, with certain modification in its parameterization schemes, we have carried out numerical simulations over the Odisha region for a 14-year period (2000–2013) to assess the malaria transmission dynamics. Few variables like temperature, rainfall and population density are provided to run VECTRI model. There is also a suite of constants that are modified to produce reliable and realistic estimates of the parameters related to the malaria vector and dynamics of malaria transmission.

## Data and Methods

### Study area

**Figure 1:**
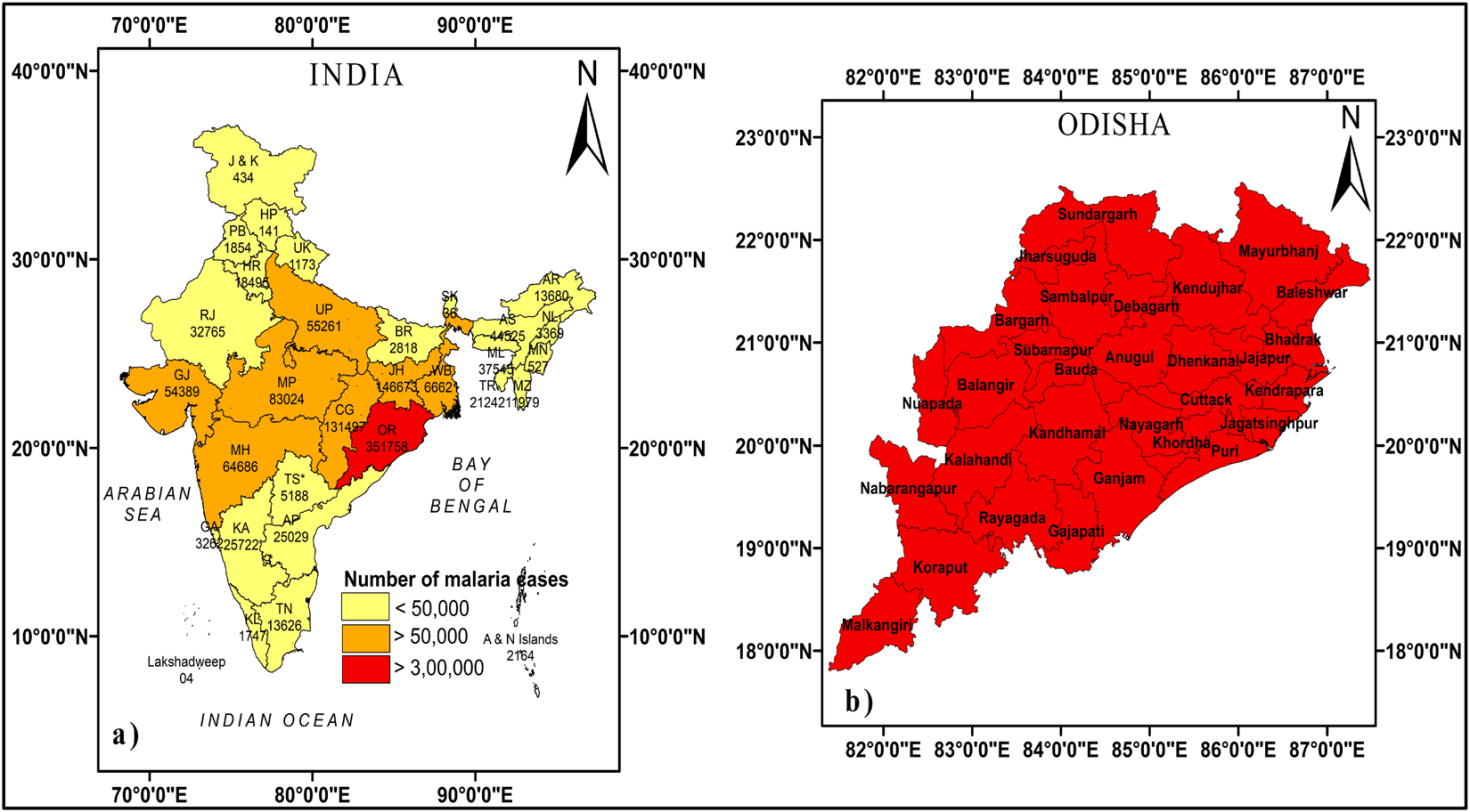
Study Area. (**a**) State wise number of malaria cases in India during 2007–2017, highlighting the study area (Odisha), (**b**) names of districts in Odisha.

This study is focused one region of interest in this study is the state of Indian state of Odisha, that is situated between 17.7°N and 22.73°N latitudes and 81.37°E to 87.53°E longitudes along the eastern coast of India and adjacent to the northern Bay of Bengal. It is likely to be one of the most vulnerable states of the country affected by the effect of climate change with respect to the occurrence of tropical cyclones, heat waves, floods, and droughts and subsequently their impact on the food supply, health, economy, and livelihood of its habitants. There is a growing concern about the changing nature of some of the vector-borne diseases at the backdrop of this changing climate. Specifically, the state has been afflicted with high incidences of malaria cases and more than 20% of the total death cases due to malaria in India occurred in Odisha during 2005 to 2010^21^. Utilizing the data (2007–2017) collected from NVBDCP for the different states in India, a map showing the number of malaria cases over each state and highlighting the study area (Odisha) is prepared and is shown in Fig. 1(a), and the individual district names in Odisha are shown in Fig. 1(b).

**Figure 2:**
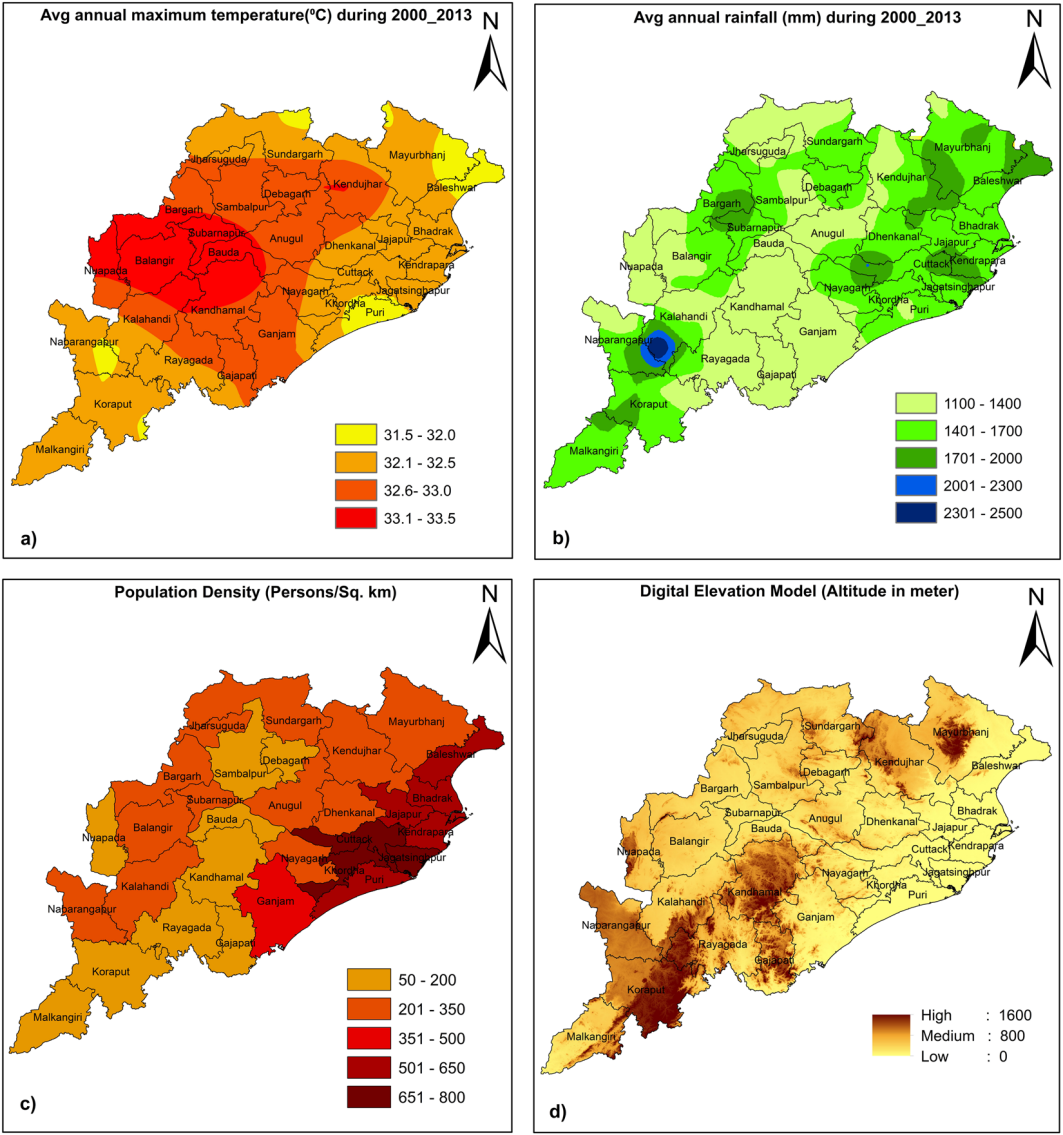
(**a**) Climatological (2000–2013) observed annual average daily maximum temperature (°C), (**b**) annual rainfall accumulation (mm), (**c**) district-wise population density as per the 2011 census, and (**d**) elevation from sea level.

The climate of Odisha is mainly of tropical type, characterized by high temperature and high rainfall. The maximum temperature in summer ranges between 35 °C to 40 °C and the total annual rainfall is 1451.2 mm. About 75% to 80% of its annual rainfall is received during June to September. More details about the climatology of Odisha are given in http://cesorissa.org/soe/Climate.pdf, a report published by the centre for environmental studies, Government of Odisha. The present climatology of Odisha in terms of annual mean daily maximum temperature and annual mean rainfall spatial distribution during the period 2000 to 2013 (14 years average) is shown in Fig. 2(a). It has been observed from the figure that the heaviest rain occurs over the northern parts of Odisha and along with the mountainous southern parts. In contrast, the central part of Odisha receives low annual average rainfall. Further, population density distribution (district wise) across the state as per the 2011 census data and topographic information is shown in Fig. 2(b). The population density of the state is 269 persons per square kilometer as per the 2011 census data. Based on the population map, the northeastern parts of the state consisting of districts Cuttack, Khorda, Jagatsinghpur are the most populated areas. Elevation is shown in Fig. 2(d). The elevation in the study area ranges from 1600 meter at the southern part to zero (at sea level) in the coastal regions.

### VECTRI model simulation

We have used VECTRI (VECToR borne disease community model of International Centre for Theoretical Physics, TRIeste) for this study, which is a dynamical malaria model developed at ICTP Italy. The model’s physics and associated parameters are based on the life cycles of the key vector, namely, *Anopheles gambiae* and parasite *Plasmodium falciparum*. The model explicitly resolves the growth stages of the egg-larvae-pupa cycle in addition to the gonotrophic and the sporogonic cycles using an array of bins for each process. The larvae are divided into a series of bins representing the fractional development state where each bin represents the density of larvae in a specific fractional growth stage. The vector state is then divided into two stages: the egg development within the female and the infective state. All vectors are advanced in terms of the egg development stage and once the vectors reach the final bin they are infective to humans. A third array of bins maps the status of the disease in the human host population, with the first bin representing the uninfected population. The explicit interaction between vector and host, the underlying numerical structure and the model architecture is described in detail in the cited reference^15^. One key feature of the VECTRI model is that it explicitly accounts for the human population density in the calculation of biting rates and host-to-vector and vector-to-host transmission probabilities for the parasite^20^. Also, using this model, impact of temperature, rainfall and population density on the transmission dynamics of malaria can be identified. VECTRI in its current set up (Version v1.3.5) is used over Odisha to model the malaria transmission of the *Plasmodium falciparum* parasite that is transmitted through the *Anopheles culicifacies* vector. The modification of parameters includes the changes in the biology of the species life cycle and changes in the climatic factors that influence the survival of the species.

In our experiment, the values of parameters such as maximum and minimum temperatures for larvae survival, time for egg hatching and pupae stages, minimal daily survival of larvae after intense rainfall, threshold temperature for egg development in vector, threshold temperature for parasite development, degree days for parasite development, etc. are modified and the constants are tuned based on the available literature for Odisha^21–24^. The list of parameters with their description and values are shown in Table 1. For this study, the India Meteorological Department (IMD) daily maximum and minimum temperature at 1° × 1° resolution^25^ and rainfall at 0.25° × 0.25° resolution^26^ from 2000 to 2013 are used as inputs to the VECTRI model for the simulation. In the simulation, the population density is prescribed as 269 (as per the 2011 census data). We have focused only on the following few variables for our analysis: rainfall (mm day^−1^), mean temperature (°C), adult mosquito density or vector density (per square meter) and entomological inoculation rate (EIR) in number of infective bites per person per day. To examine the influence of temperature and rainfall on vector density and EIR, the correlation coefficients between temperature and rainfall with vector density and EIR are computed. Estimating the EIR, the product of the vector biting rate and the sporozoites rate assesses dynamics of malaria transmission. Our analysis includes annual time series, monthly and seasonal distributions for the period 2000 to 2013 to estimate the dynamics of malaria transmission. The land surface topographic information is provided through ASTER GLOBAL DEM v2 dataset downloaded from the USGS website (https://earthexplorer.usgs.gov/). ASTER GLOBAL DEM v2 data (spatial resolution approx. 30 m) is used to produce the Digital Elevation Model map.

**Table 1:** List of parameters with their modified values in the VECTRI model set up for the Odisharegion (“–” symbol indicates unit less).

Parameter	Description	Value	Unit
neggmn	Eggs laid per female vector	110	—
rlarv_tmax	Maximum temperature for larvae survival	34	°C
rlarv_tmin	Minimum temperature for larvae survival	18	°C
rlarv_eggtime	Time for egg hatching	1	Days
rlarv_pupaetime	Time for pupae stages	4	Days
rlarv_flushmin	Minimal daily survival L1 larvae after intense rainfall	0.4	—
rlarv_flushtau	Exponential decay of flushing with rain rate	20	mm day^−1^
rtgono	Threshold temperature for egg development in vector	8	°C
dgono	Degree days for egg development in vector	35	Days
rtsporo	Threshold temperature for parasite development	16	°C
dsporo	Degree days for parasite development	111	Days
rpop_death_rate	Fraction of population renewed each year	0.008	—
rzoophilic_min	Minimum anthropophilic biting rate	0.2	—

## Results and Discussion

In the following we present analysis of annual, monthly and daily variations of rainfall, mean surface temperature, mosquito population and EIR with an objective to understand transmission dynamics of malaria in Odisha.

### Annual variations

**Figure 3:**
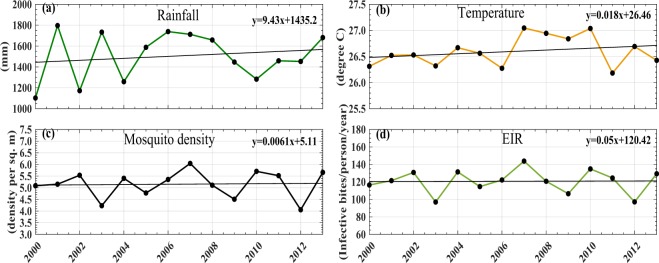
Trend in VECTRI-simulated annual mean. (**a**) Rainfall accumulation, (**b**) surface mean temperature, (**c**) mosquito density, and (**d**) EIR, from 2000–2013 over Odisha.

Figure 3 shows the temporal trends in the simulated model parameters for the period 2000–2013. In this case, all the data points on climatic factors, mosquito population and EIR are aggregated for the whole of Odisha. Results from the annual trends for different parameters show that temperature and rainfall have a linear increasing trend of 0.018 °C/year and 9.43 mm/year, respectively, during the study period. Rainfall shows a maximum value of around 1800 mm during 2001 and temperature shows a maximum value of around 27 °C during 2007 and 2010. Not many changes are observed in vector density and EIR in the state during the study period except in the years 2002, 2004, 2007 and 2010. Moreover, vector density values and EIR values vary between 40 to 60 (per sq meter) and 80 to 150 (number of infective bites per person per year), respectively.

### Monthly variations

**Figure 4:**
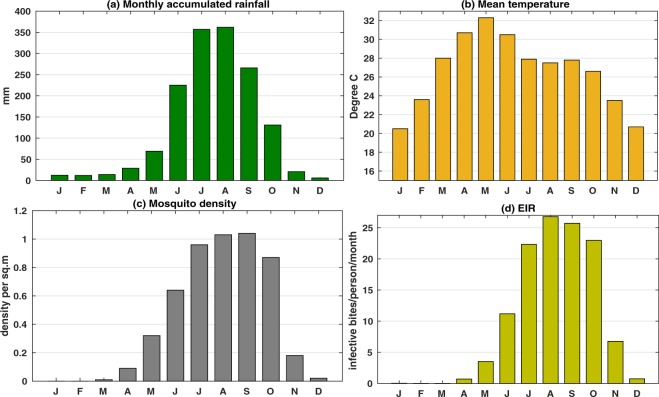
Monthly variations in VECTRI-simulated. (**a**) Accumulated rainfall, (**b**) surface mean temperature, (**c**) mosquito density, and (**d**) EIR, over Odisha. The graphs correspond to the averaging period of 2000–2013.

To establish the conditions conducive to malaria incidence and to predict the transmission of malaria peak months across the state, the monthly values for rainfall, temperature, mosquito density and EIR during the study period is shown in Fig. 4. It is found that the monthly rainfall accumulation over Odisha during the monsoon season is in the range of 200–360 mm; 10–70 mm during pre monsoon season (March to May), and 100–130 mm during post monsoon season (October and November). Again, the monthly mean surface temperature is found to be above 30°C during March to May and within 20–28 °C during other months. It has been identified that on an average the adult mosquito density is higher (0.3 to 1 per square meter) during June to October. Hence, the maximum values of mosquito density during the monsoon season coincide with the high EIR (11 to 25 infective bites/person/month). The highest level of transmission is seen during August as EIR values are highest during this month. As can be seen from the figure, malaria vector species do not persist from peak winter to the arrival of spring (December–February), but get well established during rainy season (June to September). The highest malaria vector density is observed during July to September. However, the activity declines rapidly around November.

**Figure 5:**
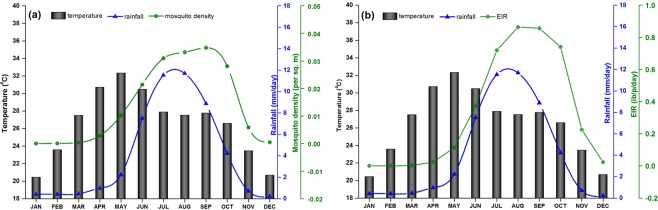
Graph showing the relationship between VECTRI-simulated surface mean temperature and rainfall with: (**a**) mosquito density, and (**b**) EIR, over Odisha. The graphs correspond to the averaging period of 2000–2013.

Further, the relationship between temperature and rainfall with vector density and EIR in terms of monthly variations for the study period is shown in Fig. 5. It is found that the relationship among temperature and rainfall with vector density and EIR are strongly non linear. During monsoon season (June to September), the values of vector density increase with temperature and rainfall. A strong correlation is seen between EIR and temperature and rainfall during the monsoon season. we find that during monsoon season, mosquito density values increase when temperature and rainfall values remain within the range 28–29 °C and 13 mm day^−1^, respectively, but above a certain threshold (temperature above 30 °C and rainfall above 13 mm day^−1^) the mosquito density values decrease.

To study the effect of rainfall and temperature on vector density and EIR, we have computed the correlation coefficient values for the whole of Odisha (figure is not shown). We found a strong relationship between daily rainfall and mosquito density with a correlation coefficient of 0.7. Similarly, a significant correlation was found between daily mean temperature and mosquito density with a correlation coefficient of 0.47. In addition, we found that the correlation coefficient values for EIR with temperature was 0.33 and with rainfall it was 0.64, thus confirming a reasonably strong relationship between EIR and the two climate variables.

### District-wise spatial variations

**Figure 6:**
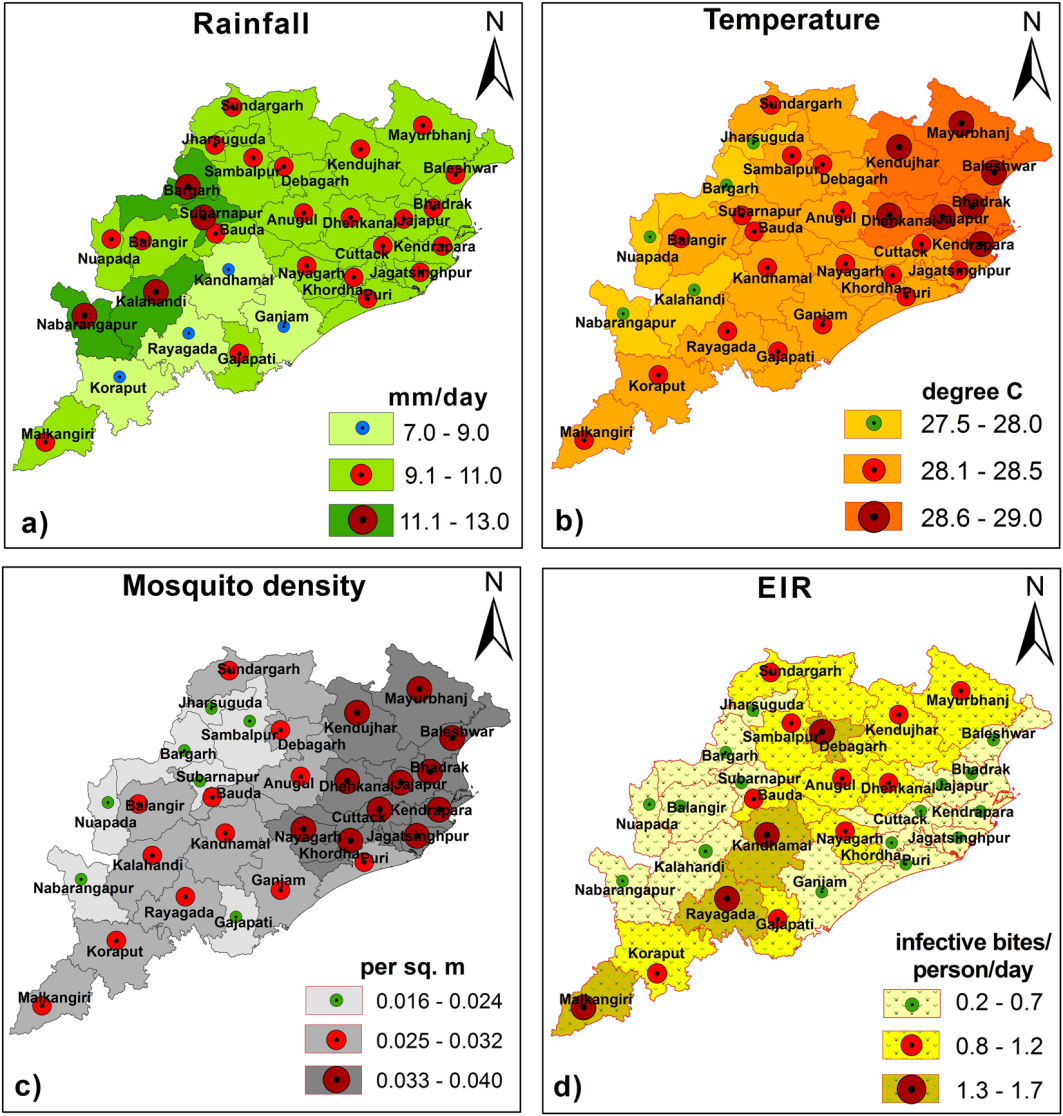
Spatial distribution of VECTRI-simulated. (**a**) Rainfall (mm day^−1^), (**b**) surface mean temperature (°C). (**c**) Mosquito density (per sq. meter), and (**d**) EIR (infective bites per person per day), during different seasons (pre-monsoon-MAM, monsoon-JJAS and post monsoon-OND), over Odisha (district wise), to understand the effect of seasons on malaria transmission. The maps correspond to the averaging period 2000 to 2013.

**Figure 7:**
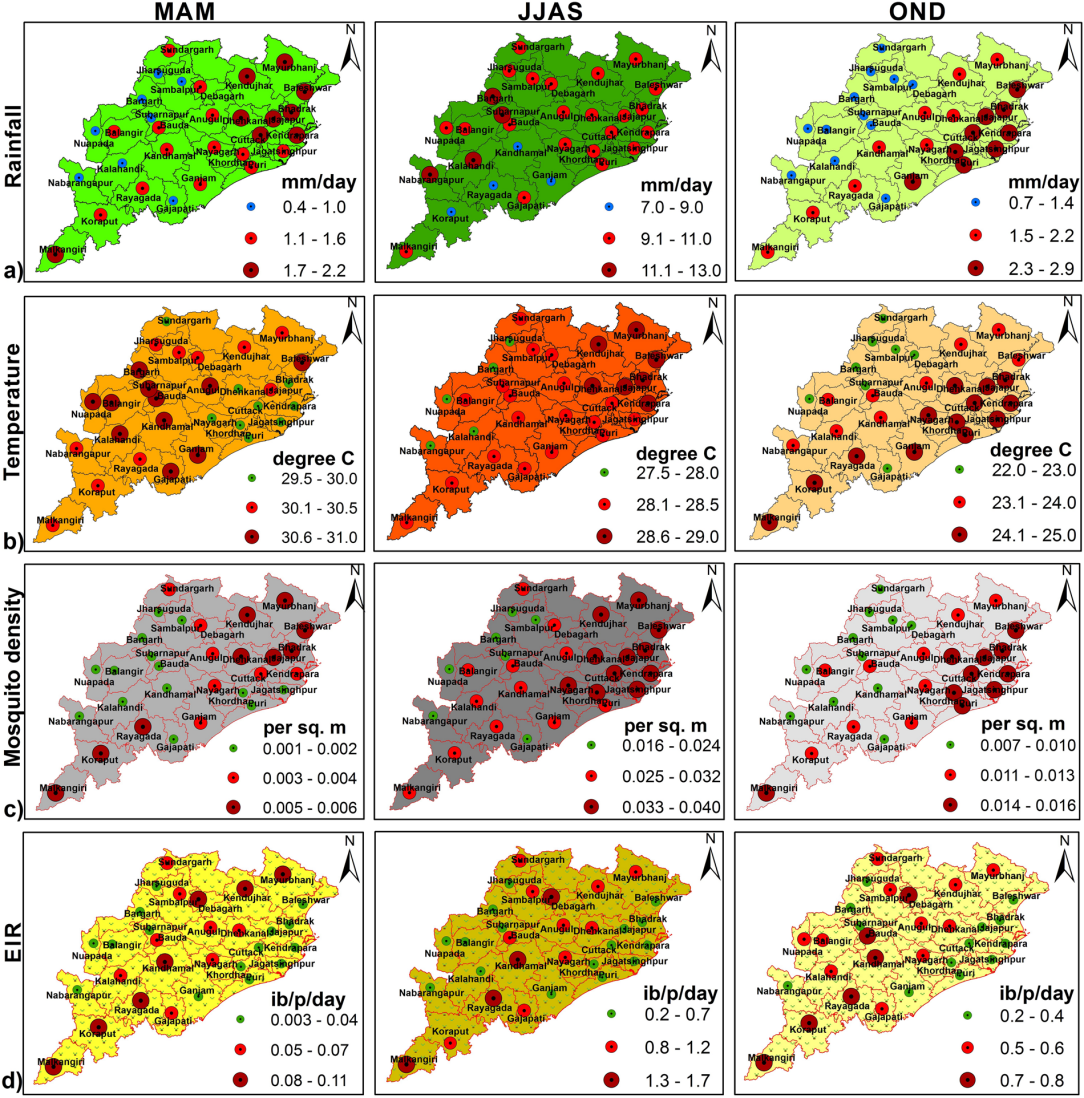
As in Fig. 6 but for summer monsoon season (JJAS).

We found that for *An*. *Culicifacies*, on an average, simulated EIR and vector density show maximum values during summer monsoon season (June to September, JJAS) as compared with the other seasons like pre monsoon season (March to May, MAM) and winter or post monsoon season (October to December, OND). Spatial distributions of the model predicted parameters during the three seasons over all districts in Odisha with high malaria incidences are shown in Fig. 6. Based on EIR estimates, it is found that JJAS season seems to be more favorable when compared to MAM and OND season over Odisha for malaria transmission. It is also seen that mosquito transmission during OND is comparatively less than the other two seasons. By considering the maximum values for each parameter, spatial patterns of rainfall, temperature, mosquito density and EIR from the VECTRI model are plotted in Fig. 7 for the monsoon season (JJAS). Our analysis for the averaged period 2000 to 2013 at the district levels indicates that few of the western districts such as Nabarangapur, Kalahandi, Bargarh, Subarnapur receive more amount of rainfall (9 to 13 mm day^−1^) during the monsoon season. Temperature over these districts ranges from 27.5 to 29 °C. The highest mosquito density is seen over most of the northern and central parts of Odisha including the districts Koraput, Kandhamal, Ganjam, Nayagarh, Khorda, Puri, Jagatsinghpur, Cuttack, Kendrapara, Dhenkanal, Jajpur, Bhadrak, Balasore, Keonjhar, Mayurbhanj, Kendujhar during the study period. Most of these districts are coastal districts and have more forest covers. It is noticed that the rainfall of 7 to 11 mm day^−1^ over these areas creates favorable conditions required for breeding of the mosquitoes larvae and parasite development. Also, the optimum temperature in which development of larvae is favored is found to be 28 to 29 °C which is within the range that supports higher malaria prevalence. Again, the malaria vector population size is higher, causing an increase in the EIR values in these areas. EIR values are found to be high over parts of central, northern and southern Odisha. The eight coastal districts (Kendrapara, Jagatsinghpur, Puri, Cuttack, Balasore, Bhadrak, Khorda, Jajpur) and few western districts (Nabarangapur, Kalahandi, Subarnapur, Bargarh) which receive maximum rainfall during the monsoon season show low malaria endemicity. EIR seem to be highest over Malkangiri, Rayagada, Kandhamal and Debgarh. These districts are predominantly inhabited by tribes and are of hilly or forest ecotypes. Our results are in agreement with the previous malaria studies which show that these districts have been more seriously affected by malaria^19,27^.

## Summary

In this study we find that temperature and rainfall are two key climate drivers that influence malaria vector development, and play a key role in characterizing the intensities of malaria transmission in the state. Mosquito species (*plasmodium falciparum*) parasite development is linked with temperature and rainfall variations. The abundance of the mosquito populations in terms of malaria vector density showed a significant strong positive correlation with temperature and rainfall. Rainfall of 7 to 11 mm day^−1^ with temperature of 28 °C to 29 °C are found to be favorable for mosquito growth, parasite development and malaria incidences during May to October over Odisha. The highest peak of malaria incidence is observed during August to September. Malaria incidences increase once the rainfall exceeds a certain threshold during the monsoon season.

Further, attempts to link temperature and rainfall to malaria vector abundance and disease incidence have yielded varied results in different districts in Odisha. Our study demonstrates that malaria transmission in Odisha shows strong spatial and seasonal variations across the districts. Specifically, based on the EIR estimates, malaria transmissions are found to be more endemic in few of the central and northern districts such as Kandhamal, Nayagarh, Bouda, Anugul, Dhenkanal, Sambalpur, Debagarh, Sundargarh, Keonjhar and Mayurbhanj, and in few southern districts such as Malkangiri, Koraput, Rayagada and Gajapati. Conversely, malaria transmission is less observed over few of the coastal and western districts of Odisha with the lower EIR values. The current tuning of the model was done for malaria occurring in 30 districts of Odisha but it would require further tuning and calibration by considering other malaria parasites and other vector species available in Odisha. VECTRI is found to be a suitable model for investigating dynamics of *An*. *Culicifacies* for different seasons over malaria endemic regions in India. However, a robust sensitivity analysis needs to be carried out for further understanding and improvement of the VECTRI model, specifically, the hydrological parameters, which are very important for the biology of *An*. *Culicifacies*.
